# Plasma from patients undergoing allogeneic hematopoietic stem cell transplantation promotes NETOSIS *in vitro* and correlates with inflammatory parameters and clinical severity

**DOI:** 10.3389/fimmu.2024.1353106

**Published:** 2024-03-14

**Authors:** Bernardo López-Andrade, Vanesa Cunill, Valero Andreu, Leyre Bento, Marina Segura-Guerrero, Andrea Moñino, Julio Iglesias, Maria Rosa Julià, Maria Antonia Durán, Maria Carmen Ballester, Josep Muncunill, Antonia Sampol

**Affiliations:** ^1^Department of Hematology, Hospital Universitari Son Espases, Palma, Spain; ^2^Department of Immunology, Hospital Universitari Son Espases, Palma, Spain; ^3^Balearic Islands Health Research Institute (IdISBa), Palma, Spain

**Keywords:** aGVHD, NETosis, NETs, IL-6, Allo-HSCT, Inflammation, Cytokines, EASIX score

## Abstract

**Introduction:**

NETosis, the mechanism by which neutrophils release extracellular traps (NETs), is closely related to inflammation. During the allogeneic hematopoietic stem cell transplantation (allo-HSCT), different stimuli can induce NETs formation. Inflammation and endothelial injury have been associated with acute graft-versus-host disease (aGVHD) and complications after allo-HSCT. We focus on the study of NETosis and its relation with cytokines, hematological and biochemical parameters and clinical outcomes before, during and after allo-HSCT.

**Methods:**

We evaluate the capacity of plasma samples from allo-HSCT patients to induce NETosis, in a cell culture model. Plasma samples from patients undergoing allo-HSCT had a stronger higher NETs induction capacity (NETsIC) than plasma from healthy donors throughout the transplantation process. An optimal cut-off value by ROC analysis was established to discriminate between patients whose plasma triggered NETosis (NETs+IC group) and those who did not (NETs-IC group).

**Results:**

Prior to conditioning treatment, the capacity of plasma samples to trigger NETosis was significantly correlated with the Endothelial Activation and Stress Index (EASIX) score. At day 5 after transplant, patients with a positive NETsIC had higher interleukin (IL)-6 and C-reactive protein (CRP) levels and also a higher Modified EASIX score (M-EASIX) than patients with a negative NETsIC. EASIX and M-EASIX scores seek to determine inflammation and endothelium damage, therefore it could indicate a heightened immune response and inflammation in the group of patients with a positive NETsIC. Cytokine levels, specifically IL-8 and IL-6, significantly increased after allo-HSCT with peak levels reached on day 10 after graft infusion. Only, IL-10 and IL-6 levels were significantly higher in patients with a positive NETsIC. In our small cohort, higher IL-6 and IL-8 levels were related to early severe complications (before day 15 after transplant).

**Discussion:**

Although early complications were not related to NETosis by itself, NETosis could predict overall non-specific but clinically significant complications during the full patient admission. In summary, NETosis can be directly induced by plasma from allo-HSCT patients and NETsIC was associated with clinical indicators of disease severity, cytokines levels and inflammatory markers.

## 1 Introduction

Allogeneic hematopoietic stem cell transplantation (allo-HSCT) represents a curative therapeutic approach for a myriad of hematological and non-hematological disorders. The downside is that it causes considerable treatment-related toxicity and non-relapse mortality (NRM) mainly due to infections and graft-versus-host disease (GVHD) (1).

The delicate balance between graft and host immune responses often results in a complex interplay that, if lost, can lead to different complications. Interaction between donor and recipient immune systems, cytokine cascades, endothelial and tissue damage and genetic predisposition are involved in the complex physiopathology of acute (a)GVHD (2). Pretransplant conditioning regimen leads to the release of inflammatory cytokines such as tumor necrosis factor-α (TNF-α), interleukin (IL)-1 and IL-6, among others which play a role in the activation of donor-derived T cells. These activated T cells release more cytokines including interferon (IFN)-gamma or IL-2 and recruit immune effector cells causing inflammation, tissue damage and a positive feedback loop that amplifies the immune response (3).

Innate immunity is crucial in the defense against infections after allo-HSCT and also, in biological reactions leading to GVHD (4). Neutrophils are the most abundant leukocytes in circulation and they are the first line of defense against pathogens. They circulate in the bloodstream and migrate to the tissue upon injury or infection. Three main antimicrobial functions have been identified for neutrophils: phagocytosis, degranulation, and the recently characterized, NETosis. NETosis was described by Takei et al. (5) as a programmed cell death in which the neutrophils release extracellular traps (NETs). NETs are structures composed of DNA and modified chromatin which also contain cytotoxic and bactericidal enzymes like myeloperoxidase (MPO) among other proteases and lysozymes. NETs bind and kill pathogens, but excessive NETosis could be detrimental to the host, as they have been related to cardiovascular, autoimmune, inflammatory and oncological diseases (6). They are also involved in different inflammatory processes, thrombogenesis and endothelial dysfunction (7).

In recent years, evidence has accumulated that endothelial dysfunction in the context of inflammation and cytokine dysregulation is one of the mechanisms described in aGVHD and contributes to severe complications after allo-HSCT (8, 9). In preclinical models, vulnerability biomarkers and ongoing endothelial tissue injury markers appear to be a high-priority goal to reduce the risks of allo-HSCT (10). Approaches using flexible laboratory and clinical variables have been assessed to predict complications during allo-HSCT. These variables such as ferritin (11) and other surrogate markers of inflammation have been used to calculate scores like the Endothelial Activation and Stress Index (EASIX) or the Modified-EASIX (M-EASIX) (12, 13). Both scores, first introduced to predict the cytokine release syndrome during chimeric antigen receptor (CAR) T cell therapy, seek to determine inflammation and endothelium damage. However, biomarkers for predicting post-allo-HSCT survival, NRM and, especially, for aGVHD remain not well defined or used in clinical practice and this represents a clinical challenge.

In the present study, we focused on the study of NETosis during allo-HSCT and its potential relation with cytokines, hematological and biochemical parameters and clinical outcomes. Understanding the biological mechanisms that underlay the inflammatory response during allo-HSCT would allow the use of new biomarkers and guide personalized therapeutic options.

## 2 Methods

### 2.1 Patient cohort

A total of 19 adult patients who underwent allo-HSCT between January 2019 and January 2020 in Son Espases Hospital (HUSE) were recruited for this study. The study was conducted according to the ethical guidelines of the 1975 Declaration of Helsinki and approved by CEIC (Balearic Islands Clinical Research Ethics Committee). Written informed consent to have their clinical information published in medical or scientific journals was obtained from all subjects.

Different baseline diagnoses and indications for allo-HSCT were enrolled in this real-world study. All the diagnoses were evaluated according to the World Health Organization 2016 criteria (**Table 1**).

**Table 1:** Cohort demographics and clinical characteristics of the patients who received an allo-HSCT.

Subject ID	Age	Sex	Prior therapy lines	Indication for allo-HSCT	Therapy before allo-HSCT	Response prior allo-HSCT	Conditioning	Stem cell source	Donor
HSCT 1	25	F	1	T- ALL	PETHEMA LAL-T	CR MRD-	MAC	PB	Unrelated donor (10/10)
HSCT 2	60	F	1	ALL Ph+	PETHEMA LAL Ph+	CR MRD-	MAC	PB	Related donor (haploidentical)
HSCT 3	68	M	0	HR-MDS	None	SD	RIC	PB	Unrelated donor (10/10)
HSCT 4	56	M	1	HR-MDS	AZA+VEN	CR	RIC	PB	Related donor (identical)
HSCT 5	18	F	1	AA	ATG + corticosteroids	SD	RIC	BM	Unrelated donor (9/10)
HSCT 6	58	M	1	CMML	AZA	CR	RIC	PB	Unrelated donor (10/10)
HSCT 7	67	M	1	HR-MDS	AZA	SD	MAC	PB	Related donor (haploidentical)
HSCT 8	60	M	1	sMF	RUX	SD	RIC	PB	Related donor (identical)
HSCT 9	55	M	0	MPN Unclassifiable NPM1+	None	SD	RIC	PB	Unrelated donor (9/10)
HSCT 10	51	M	3	AML FLT3+	Gilteritinib + Venetoclax	PR	MAC	PB	Related donor (identical)
HSCT 11	55	F	1	CMML	AZA	SD	RIC	PB	Unrelated donor (10/10)
HSCT 12	51	M	1	AML	CETLAM<70	CR	MAC	PB	Related donor (identical)
HSCT 13	57	M	1	CMML	AZA + RUX	SD	RIC	PB	Unrelated donor (10/10)
HSCT 14	59	F	1	HR - MDS/MPN	IDA + Ara-C	SD	RIC	PB	Unrelated donor (9/10)
HSCT 15	47	M	1	CML Blast phase	CETLAM<70 Dasatinib	CR	MAC	PB	Related donor (identical)
HSCT 16	52	M	2	AMML	FLAG-IDA	CR	MAC	PB	Related donor (identical)
HSCT 17	69	F	0	HR-MDS	None	SD	RIC	PB	Unrelated donor (10/10)
HSCT 18	49	F	2*	sAML*	FLAG-IDA	CR	RIC	PB	Unrelated donor (10/10)
HSCT 19	48	M	0	HR-MDS	None	SD	RIC	PB	Unrelated donor (10/10)

allo-HSCT, allogeneic hematopoietic stem cell transplantation; ATG, anti-thymocyte globulin; AZA, azacitidine; VEN, venetoclax; RUX, ruxolitinib; CR, complete response; PR, partial response; SD, stable disease; BM, bone marrow; PB, peripheral blood; RIC, reduced intensity conditioning; MAC, myeloablative conditioning; TBI, total body irradiation; AML, acute myeloid leukemia; ALL, acute lymphoblastic leukemia; Ph+, Philadelphia positive; AA, aplastic anemia; AMML, acute myelomonocytic leukemia; CMML, chronic myelomonocytic leukemia; HR-MDS, high-risk Myelodysplastic syndrome; sAML, secondary acute myeloid leukemia; CML, chronic myeloid leukemia; sMF, secondary myelofibrosis; MPN, myeloproliferative neoplasm; *Relapse after prior allo-HSCT.

Peripheral blood from patients was collected in heparin (for plasma extraction) and BD vacutainer (serum extraction tubes) and immediately processed, upon the arrival of the patients to the center before the start of the conditioning regimen (D-1), on infusion (D0) and every 5 days afterward (D5, D10, D15, D20 and D30), if available. A total of 107 serum and plasma samples were collected and stored at -70°C until further use. Demographic and clinical data were recorded, as well as, routine laboratory tests including coagulation and inflammatory markers like ferritin or C-reactive protein (CRP). These variables were used to calculate EASIX and m-EASIX prognostic scores.

### 2.2 Isolation of neutrophils from peripheral blood samples and cell culture

Neutrophils were isolated from the peripheral blood of healthy donors using dextran sedimentation. Briefly, peripheral blood collected in heparin tubes was incubated with 6% dextran for 45 min at 37°C in a 5% CO2 atmosphere. After sedimentation, neutrophil-rich supernatant at the upper layer was collected in a sterile tube, centrifuged and washed with RPMI-1640 medium. Red blood cell (RBC) lysis was performed using an RBC lysis buffer (1 mL of physiological saline solution plus 3 mL of distilled water) and vortex for 30 seconds. Afterward, 1 mL of 3.5% saline solution was added and centrifuged for 5 minutes. This process was repeated three times.

**Figure 1:**
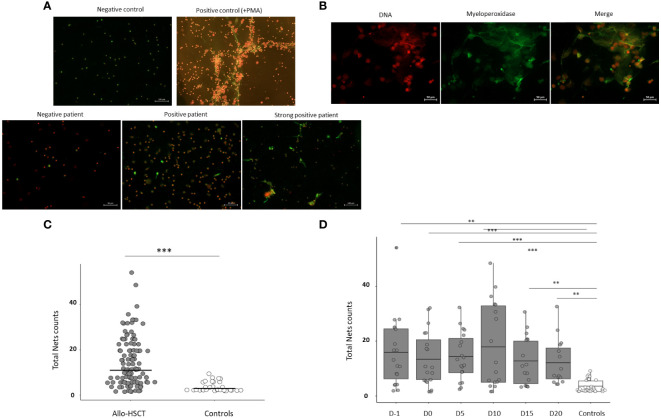
Assessment of the capacity of plasma samples from allo-HSCT patients to induce NETs formation, *in vitro*. **(A)** Representative images from patients and controls. **(B)** Iodide solution (right panel) and anti-MPO-FITC (central panel) images merged in the left panel to demonstrate co-localization of MPO and extracellular DNA ejected in cells undergoing NETosis. **(C)** Comparison of total NETs counts between allo-HSCT patients and healthy controls. Total NET counts were calculated as the sum of NETs induced by plasma samples in the 10-field counts. **(D)** Comparison of total NETs counts induced from allo-HSCT patient samples at different time points studied (D-1, D0, D5, D10, D15 and D20) and healthy controls. Each dot represents one individual patient sample and lines represent the average. Data are given as mean (T-test and ANOVA tests. p-values: **p<0.01, ***p < 0.001).

Isolated neutrophils were suspended in RPMI-1640 medium supplemented with 10% heat-inactivated fetal calf serum, glutamine (2 mM) and antibiotics (penicillin and streptomycin). Samples were filtered to remove cell accumulations using 50 µm pore size filters. Then, cells were cultured (2×10^6^ cells/mL) in Millicell EZ SLIDE glass (Millipore, Merck KGaA) and each well was incubated with different patients’ plasma samples. Negative and positive controls, stimulated with phorbol 12-myristate 13-acetate (PMA; 75 ng/mL) and ionomycin (1.5 ug/mL) (both from Sigma-Aldrich), were also prepared in each experiment (**Figure 1A**). Cultures were maintained for 4 hours at 37°C in a 5% CO2 atmosphere.

### 2.3 Immunofluorescence

A staining protocol was used to evaluate NETosis, *in vitro*, by using fluorescent microscopy (Nikon Eclipse E400). Briefly, after incubation, neutrophils were fixated with 2% of paraformaldehyde and stained with Iodide solution (1.0 µg/ml Merck Life Science S.L.) and anti-myeloperoxidase (MPO)-FITC (Milteny Biotec, REAfinity™, USA) for extracellular DNA and MPO identification. Immunofluorescence images were independently interpreted by two observers, who were blinded to the sample identity and any discrepancy was solved by consensus. For each sample, 10 fields were counted and the number of visible NETs in each one was registered. The median count from both observers was used for the analyses. The fluorescent microscopic evaluation and quantification of positive NETs were carried out at 20x magnification. Inconclusive results were solved by 110x magnification with immersion oil. Neutrophils were considered positive, for NETs formation, according to single-cell morphology (nuclear loss of lobules and ejection of DNA from the cell membrane) together with positive double immunolabeling of NET structures using anti-MPO and propidium iodide solution (co-localization of DNA and MPO) (**Figure 1B**).

### 2.4 Cytokine quantification

The Human Inflammatory Cytokine kit BD Cytometric Bead Array assay (BD Biosciences, U.S.A) was used to quantify serum concentrations of IL‐6, IL‐8, IL‐10, TNF-α and IL-12p70 by flow cytometry (FACS Verse BD Biosciences, U.S.A), according to the manufacturer´s instructions.

### 2.5 ELISA of citrullinated histone 3 (H3Cit)

The serum concentration of Citrullinated histone 3 (H3Cit) was measured using a sandwich enzyme-linked immunosorbent assay (ELISA) developed by Cayman Chemicals (Ann Arbor, MI, USA) according to the manufacturer´s instructions. This assay employed a monoclonal antibody specific for histone H3 citrullinated at R2, R8 and R17 (clone 11D3). The lower limit detection of the assay was 0.1 ng/ml.

### 2.6 Statistical analysis

The normality of the distribution was assessed using the Shapiro test. Normally distributed variables were expressed as mean (± standard deviation) and non-normally distributed data were presented as median (min-max). T-test and ANOVA tests or their non-parametric counterparts, the Mann-Whitney and Kruskal-Wallis tests, were used to compare differences between two groups or more than two groups, respectively. Correlations were analyzed using the Spearman correlation test. A p-value lower than 0.05 was considered significant.

ROC analysis was used to determine the power of variables to differentiate groups, and the area under the curve was calculated; significant cut-off levels were calculated using a Youden index. Logistic regression analysis was used to compare independent variables. ROC curves were performed using 35 negative and positive controls. Two cut-off values obtained by ROC analysis were used in the study. The cut-off value for NETS positivity was ≥ 0.85 NETs counts per field (sensitivity 100% specificity 96.77%). The second cut-off value used to identify strong NETS positivity was ≥ 2.27 NETs counts per field (sensitivity 100% specificity 100%). The statistical analysis was performed using GraphPad Prism software version 5.0. and R version 4.2.

## 3 Results

### 3.1 Clinical, functional and analytical characteristics of patients

**Table 1** summarizes the main patient and allo-HSCT information. Briefly outlined, the median age of the study cohort at the time of the allo-HSCT was 52.8 years (range 18-69) and 12 (63%) were male and 7 (37%) female. The median number of previous lines of therapy before transplant was 1 (0 to 3). Disease status before allo-HSCT was as follows: complete response (CR) in 8 patients (42%), partial response (PR) in 1 (5%) patient and stable disease (SD) in 10 (53%). Overall, 58% of patients received reduced-intensity conditioning (RIC) allo-HSCT, and most patients received grafts from related and unrelated HLA-matched donors (73%), followed by 9/10 mismatched unrelated donors (16%) and haploidentical donors (10%). A proportion of 18 grafts were obtained from peripheral blood and only 1 from bone marrow.

**Table 2** displays the complications presented by patients, divided into two periods: (i) before day 15 after transplant, referred to as early complications (58%), and (ii) during admission (75%). We considered as relevant complications: (i) aGVHD, (ii) infection, (iii) sepsis, (iv) veno-occlusive disease (VOD), hemorrhage (v) and (vi) transplant-associated thrombotic microangiopathy (TR-TMA). We did not consider mucositis as a severe complication, but it was included in the table (**Table 2**). **Table 2** also depicts patients who developed aGVHD (47%) and chronic (c)GVHD (10% of patients).

**Table 2:** Complications presented by patients.

Subject ID	Early complications	Complications during admission	GVHD		Relapse <100days
			<100 days (aGVHD)	>100 days (cGVHD)	
**HSCT 1**	No	aGVHD	Yes (Grade II)	No	No
**HSCT 2**	No	No	No	No	No
**HSCT 3**	aGVHD, TR-TMA	TR-TMA, Infection (*Staphylococcus epidermidis)*	Yes (Grade I)	No	No
**HSCT 4**	aGVHD	Infection (*Staphylococcus epidermidis* and BK virus)	Yes (Grade II)	No	No
**HSCT 5**	Infection (*Enterococcus faecium)*	aGVHD, Infection (*Enterococcus faecium)*	Yes (Grade II)	No	No
**HSCT 6**	No (Mucositis)	No	No	No	No
**HSCT 7**	CNS hemorrhage, death	CNS hemorrhage, death	No	No	No
**HSCT 8**	VOD, Infection *(Fusobacterium nucleatum)*	VOD, aGVHD	Yes (Grade III)	No	No
**HSCT 9**	Infection *(Staphylococcus epidermidis)*	Infection *(Staphylococcus epidermidis)*, bleeding	No	Overlap (moderate GVHD)	No
**HSCT 10**	Infection *(Escherichia coli)*	Infection *(Escherichia coli)*	No	No	Yes
**HSCT 11**	Sepsis (*Pseudomonas aeruginosa*), death	Sepsis (*Pseudomonas aeruginosa*), death	No	No	No
**HSCT 12**	No (Mucositis)	Infection *(Escherichia coli)*	Yes (Grade I)	No	No
**HSCT 13**	No (Mucositis)	Infection *(Staphylococcus epidermidis)*	No	No	Yes
**HSCT 14**	Infection *(Escherichia coli)*	Infection *(Escherichia coli)*	Yes (Grade III)	No	No
**HSCT 15**	No	Infection *(Staphylococcus epidermidis)*	No	Moderate cGVHD	No
**HSCT 16**	No	Infection *(Stenotrophomonas maltophilia)*	Yes (Grade III)	No	No
**HSCT 17**	Infection *(Corynebacterium tuberculostearicum)*	Infection *(Corynebacterium tuberculostearicum)*	Yes (Grade II)	No	No
**HSCT 18**	No (Mucositis)	No	No	No	No
**HSCT 19**	Infection *(Escherichia coli)*	Infection *(Escherichia coli)*	No	No	No

VOD, veno occlusive disease; TR-TMA, transplant-related thrombotic microangiopathy; aGVHD, acute graft versus host disease; cGVHD, chronic graft versus host disease; CNS, central nervous system.

Complications are classified into early complications (before day 15 after transplant) and complications during admission. Presence of graft versus host disease (GVHD) and/or relapse is also indicated.

The most frequent early complication was infection (42%) and aGVHD (10%). During admission, the most frequent associated complications were also infections (68%), of which 1 developed sepsis, followed by aGVHD (16%) (**Table 2**). Overall, during follow-up and before day 100, 47% of patients presented aGVHD; The grading for these patients was as follows: 2 (grade I), 4 (grade II) and 3 (grade III). After day 100, 1 patient had a moderate overlap GVHD and 1 patient developed a moderate cGVHD (**Table 2**).

### 3.2 Plasma from allo-HSCT induces NETosis *in vitro*

We were interested in evaluating whether plasma samples from allo-HSCT patients were able to induce NETs formation, *in vitro*, in human neutrophils from healthy controls. We studied the NETs induction capacity (NETsIC) in 107 plasma samples from 19 allo-HSCT patients. Plasma samples from allo-HSCT patients were stronger NETs inducers than plasma from healthy controls (14.4 counts vs 3.5 counts; p<0.001) (**Figure 1C**).

Then, we evaluated how the NETsIC evolved throughout the transplantation process. To do so, we compared the NETsIC of plasma samples from allo-HSCT patients between the different time points assessed (D-1, D0, D5, D10, D15 and D20) with those taken from healthy controls. Accordingly, the NETsIC of plasma samples from allo-HSCT patients remained significantly elevated compared to controls at all time points studied (D-1, D0, D5, D10, D15 and D20) (**Figure 1D**). However, regarding allo-HSCT patient samples, similar levels were found between the different time points of transplantation tested (**Figure 1D**).

**Figure 2:**
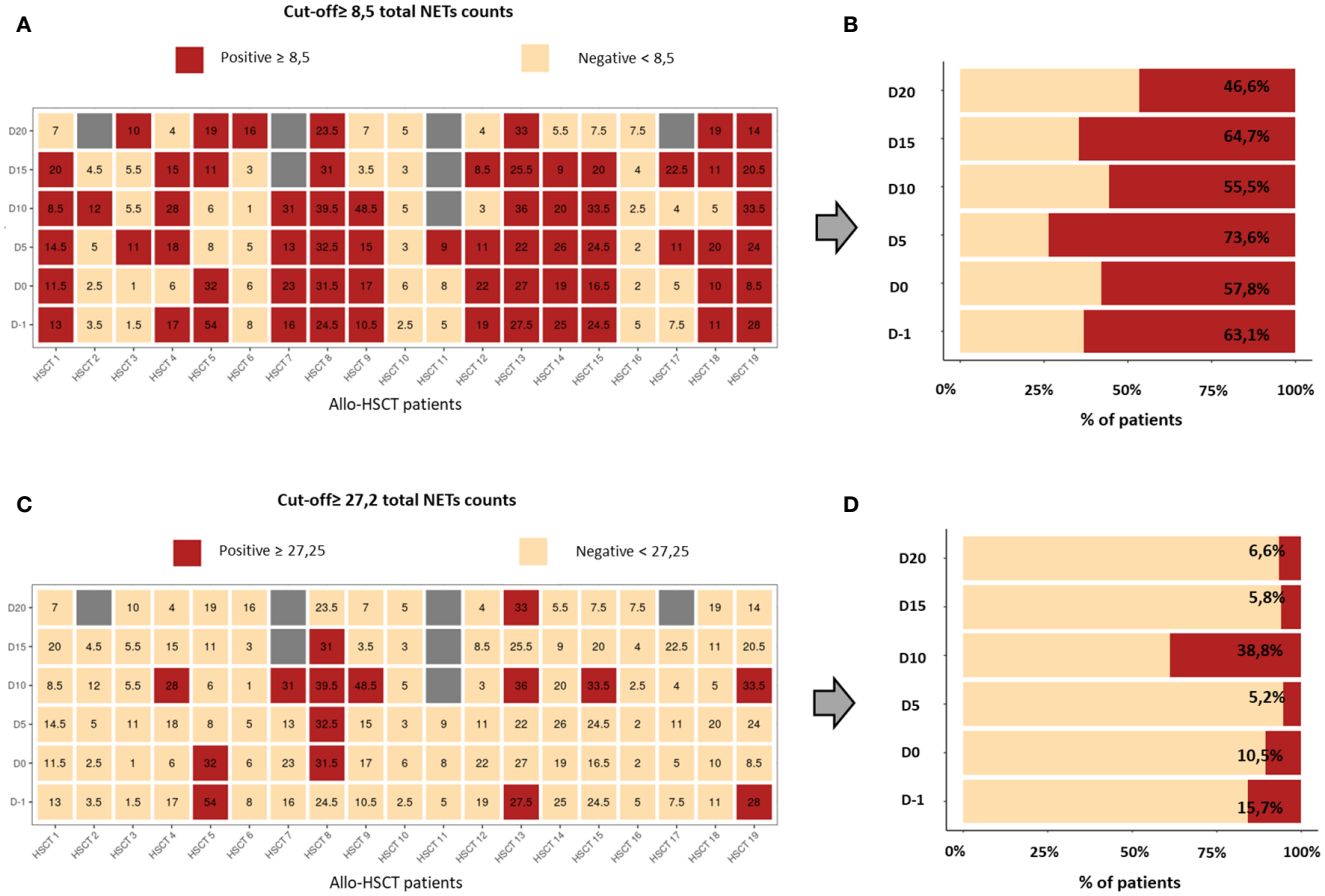
Representation of total NETs counts of all patients and at all time points evaluated. **(A)** Heat map showing, in red, the patient’s samples with a positive NETsIC and, in pale orange, samples with a negative NETsIC. **(B)** Percentage of patients who presented a positive (red) and negative (pale orange) NETsIC. The percentages of positive and negative patient groups were calculated for each of the studied times. **(C)** Heat map showing, in red, the patient’s samples with a strong NETsIC and, in pale orange, samples with a non-strong NETsIC. **(D)** Percentage of patients who presented a positive (red) and negative (pale orange) strong-NETsIC. The percentages of positive and negative patients were calculated for each of the studied times.

If we focused particularly on each time point evaluated, we observed that total NETs counts were highly variable between patients (**Figure 1D**). Therefore, using ROC analysis we established two cut-off values. The first one (≥8.5 total NETs counts) was used to discriminate between patients whose plasma was able to trigger NETosis (NETs+IC group) and those who did not (NETs-IC group) (**Figure 2A**). The second cut-off value (≥ 27.2 total NETs counts) was established to identify allo-HSCT samples with a strong NETsIC (**Figure 2C**). As depicted in **Figure 2B**, in all time points evaluated over half of the plasma samples were capable of inducing NETosis. Remarkably, at D5 after graft infusion, 73% of the patient´s plasma was able to induce NETosis. These results suggested that most of the allo-HSCT patient’s plasma could induce NETosis throughout the transplantation process. Interestingly, when we examined which samples had a strong NETsIC we found that the maximum was reached at D10 after transplant with 38.8% of positive samples (**Figure 2D**).

To rule out that treatments previous to the conditioning regimen may have influenced the ability of plasma samples to induce NETosis, the NETsIC was compared between treated and the 4 untreated patients (**Table 1**), and no statistically significant differences were found (**Supplementary Table S1**).

### 3.3 Maximum IL-6 and IL-8 levels were reached at day 10 after allo-HSCT and NETosis was associated with IL-6 and IL-10 serum levels

**Figure 3:**
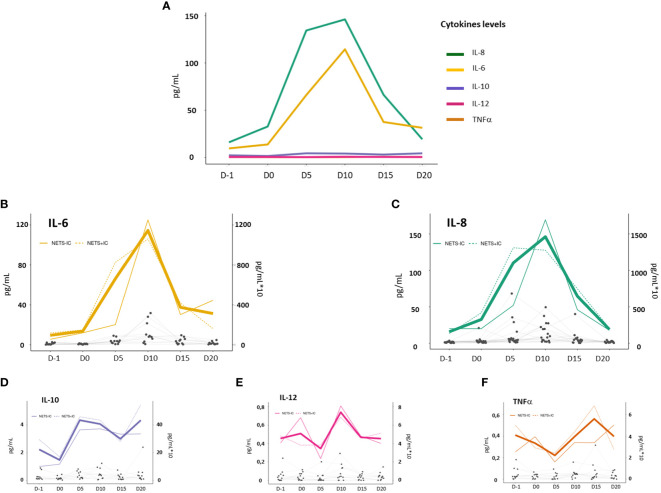
Cytokine levels in allo-HSCT patients before (D-1) and after allo-HSCT (D0, D5, D10, D15 and D20). **(A)** Median serum concentration (pg/ml) of IL-8 (green line), IL-6 (yellow line), IL-10 (purple line), TNF-α (orange line) and IL-12p70 (pink line). **(B–F)** The bold color line shows the mean cytokines levels of serum samples from allo-HSCT patients (results are shown on an x10 scale). Dotted and continuous color lines represent the mean cytokines levels of NETs+IC and NETs-IC patient groups, respectively (scale x10). Gray dots represent an individual patient sample and successive repeated measures on the same individual are represented by connecting lines.

Multiple NETs inducers including cytokines such as IL-6 or IL-8 have been described (14, 15), Thus, we quantified serum levels of IL-8, IL-6, IL-10, TNF-α and IL-12p70 in the 107 patient’s serum samples before, during and after allo-HSCT. It is noteworthy, that IL-8 followed by IL-6 experienced the largest increases in allo-HSCT patients during the evaluated period (**Figure 3A**). Indeed, these two cytokines presented a similar profile: peak levels were reached at D10 after stem cell infusion and returned to pretransplant values, approximately, at D20 after infusion. Specifically, when we performed a longitudinal analysis, the highest differences were found between D-1 and D10 for IL-6 (8.17 pg/ml vs 119.4 pg/ml p<0.001) and IL-8 (16.06 pg/ml vs 168.1 pg/ml p<0.001).

Serum levels of IL-10 increased slightly (p=0.07) and remained slightly elevated until D20. TNF-α and IL-12p70 levels did not increase throughout the studied period (**Figure 3A**).

**Figure 4:**
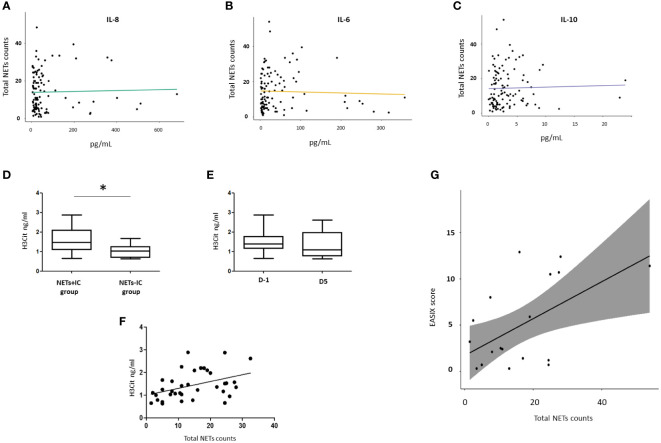
Correlation of IL-8 **(A)**, IL-6 **(B)** and IL-10 **(C)** serum levels and total NETs counts. **(D)** Comparison of H3Cit sera levels between NETs+IC and NETs-IC patient groups. **(E)** Comparison of H3Cit sera levels between D-1 and D5 after allo-HSCT. **(F)** Correlation between H3Cit sera levels (ng/ml) and total NETs counts. **(G)** Correlation between EASIX score and total NETs count on D-1 (Prior to conditioning regimen). In the correlation analysis, each dot represents a sample. Data are given as mean (T-test. p-values: *p<0.05).

Then, we evaluated the potential relationship between NETosis and cytokines levels in allo-HSCT settings (**Figures 3B–F**). We compared the cytokines levels between patients with positive and negative NETsIC. We only found significantly higher levels of IL-10 before transplant (2.94 pg/ml vs 1.03 pg/ml; p<0.05) and IL-6 (82.5 pg/ml vs 20.2 pg/ml p<0.05) at D5 after graft infusion in NETs+IC group compared to NETs–IC group (**Table 3**). No differences in IL-8 levels were found between patients with positive and negative NETsIC. Interestingly, as depicted in **Figure 3C**, IL-8 levels seemed to be higher in the NETs+IC patients group until D5 after transplant but this trend reversed on D10. However, no statistically significant difference was found in IL-8 levels between patients with positive or negative NETsIC. Furthermore, we did not find any correlation between cytokines levels and the NETsIC of the plasma samples from allo-HSCT patients (**Figures 4A–C**).

**Table 3:** Comparison of cytokine levels between NETs-IC and NETs+IC allo-HSCT patient groups.

Mean Cytokine Levels (pg/mL)	D-1		D0		D5	
	NETs-IC group	NETs+IC group	NETs-IC group	NETs+IC group	NETs-IC group	NETs+IC group
**IL-8**	20.1	13.4	18.7	27.14	52.04	131
**IL-6**	5.54	11.9	12	14.9	**20.2**	**82.5**
**IL-10**	**1.03**	**2.94**	1.17	1.7	3.65	4.59
**TNF-α**	0.25	0.5	0.39	0.29	0.16	0.24
**IL-12p70**	0.41	0.48	0.68	0.38	0.24	0.38

NETs-IC group, group of patients with a negative capacity to induce NETosis; NETs+IC group, group of patients with a capacity to induce NETosis in vitro; D-1, pre-conditioning; D0, infusion day; D5, day 5 after graft infusion. Data were expressed as mean. Bold values mean there is statistical significance (p<0.05).

In our cohort, cytokine levels were not affected by previous treatments to the conditioning regimen (**Supplementary Table S1**).

### 3.4 Citrullinated histone 3 (H3Cit) serum levels were higher in the NETs+IC group

Citrullinated histone 3 (H3Cit) is one of the main components of NETs structures and the evaluation of H3Cit levels is an indirect way of measuring NETosis. We determined H3Cit levels in serum samples from allo-HSCT patients previous to the conditioning regimen (D-1) and at day 5 (D5) post-transplant and correlated them with NETsIC. Higher H3Cit levels were found in the group of patients with a positive NETsIC (NETs+IC group) compared to patients with a negative NETsIC (NETs-IC group) (1.6 ng/mL vs 1.05 ng/mL; p<0.05) (**Figure 4D**). However, we did not find a correlation between H3Cit levels and NETsIC (p<0.05; r2 0.17; Sy.x 0.57) (**Figure 4F**). No differences were found when H3Cit levels were evaluated before and after allo-HSCT (**Figure 4E**).

### 3.5 NETsIC and Coagulation parameters

NETosis has been extensively related to thrombotic mechanisms (16–18). For that reason, we analyzed the coagulation parameters, prothrombin time (PT) and fibrinogen, after ruling out prior anticoagulation treatments in the patients. We did not find differences when PT and fibrinogen levels were compared between NETs+IC and NETs-IC patient groups (**Table 4**). However, when we evaluated the PT and fibrinogen levels in all the allo-HSCT samples with a strong NETsIC we found higher fibrinogen levels (744 mg/dL vs 598 mg/dL; p<0.05) and a trend toward lower PT (73.2% vs 80.2%; p=0.1) compared to samples with a non-strong NETsIC. Moreover, prior to consolidation treatment (D-1), significantly lower PT was found in patients with strong NETsIC (**Table 5**).

**Table 4:** Comparison of laboratory markers between NETs-IC and NETs+IC allo-HSCT patient groups.

Hematological and serum biochemistry parameters	D-1		D5	
	NETs-IC group	NETs+IC group	NETs-IC group	NETs+IC group
**Leukocytes** **x10^9^cells/L**	2.83 (1.25)	9.20 (19.6)	0.16 (0.18)	0.21 (0.57)
**Neutrophils**	1.67 (2.09)	6.96 (17.4)	0.09 (0.11)	0.17 (0.53)
**Hb gr/dL**	9.96 (1.54)	8.88 (2.63)	8.50 (1.26)	7.87 (1.97)
**RDW %**	15.4 (2.19)	17.3 (4.12)	12.9 (1.51)	14.3 (1.79)
**Platelets x10^9^cells/L**	113 (81.5)	81.4 (81.4)	41.4 (52.7)	14.9 (19.6)
**MPV fl**	9.81 (3.92)	9.05 (2.14)	11.5 (3.87)	20.4 (23.0)
**PDW %**	15.2 (2.39)	16.6 (2.43)	12.9 (3.14)	12.0 (1.85)
**NLR**	1.41 (1.46)	2.82 (3.00)	3.70 (3.92)	2.75 (6.12)
**CRP mg/dL**	0.41 (0.44)	1.84 (3.83)	**4.14 (2.55)**	**8.73 (5.72)**
**Ferritin ng/mL**	1444 (2589)	1953 (1732)	2948 (2850)	3881 (8866)
**Cr mg/dL**	0.76 (0.15)	0.87 (0.39)	0.65 (0.10)	0.64 (0.21)
**LDH U/L**	192 (54.0)	262 (224)	169 (52.7)	198 (86.5)
**Fibrinogen mg/dL**	460 (97.7)	518 (263)	579 (154)	569 (143)
**PT %**	85.8 (10.5)	87.1 (13.2)	83.0 (11.2)	82.5 (12.4)
**M-EASIX Score**	2.01 (3.09)	25.3 (71.8)	**118 (123)**	**375 (431)**
**EASIX Score**	2.93 (2.88)	6.03 (5.14)	14.7 (16.2)	28.6 (43.5)

NETs-IC group, group of patients with a negative NETs induction capacity; NETs+IC group, group of patients with a positive NETs induction capacity Hb, hemoglobin; RDW, red cell distribution width; MPV, mean platelet volume; CRP, C-reactive Protein; Cr, creatinine; PDW, platelet distribution width; NRL, neutrophil-to-lymphocyte ratio; PT, prothrombin time. Data were expressed as mean (± standard deviation). Bold values indicate a statistical significance (p<0.05).

**Table 5:** Comparison of laboratory markers between allo-HSCT patients with a strong NETsIC (strong NETsIC group) and patients without them (non-strong NETsIC group).

Hematological and serum biochemistry parameters	D-1		D10	
	Non-Strong NETsIC group	Strong NETsIC group	Non-Strong NETsIC group	Strong NETsIC group
**Leukocytes** **x10^9^cells/L**	7.20 (17.1)	5.02 (5.16)	0.03 (0.01)	0.02 (0.02)
**Neutrophils**	5.40 (15.2)	2.97 (3.28)	0.01 (0.00)	0.02 (0.02)
**Hb gr/dL**	**9.58 (2.39)**	**7.65 (0.53)**	7.79 (0.89)	7.83 (2.37)
**RDW %**	16.4 (3.01)	17.9 (6.74)	13.2 (1.40)	12.7 (1.67)
**Platelets x10^9^cells/L**	**105 (81.7)**	**27.7 (30.7)**	**9.53 (6.12)**	**4.60 (2.95)**
**MPV fl**	9.18 (3.05)	10.1 (1.35)	**12.5 (2.71)**	**15.9 (2.43)**
**PDW %**	16.0 (2.26)	16.2 (3.94)	12.2 (2.71)	11.7 (2.75)
**NLR**	2.30 (2.71)	2.29 (2.29)	**0.93 (0.83)**	**2.78 (2.22)**
**CRP mg/dL**	0.61 (0.68)	5.03 (7.60)	13.6 (7.27)	13.3 (6.39)
**Ferritin ng/mL**	1710 (2072)	2066 (2194)	2416 (2298)	6996 (12505)
**Cr mg/dL**	0.80 (0.32)	0.96 (0.33)	0.60 (0.14)	0.78 (0.28)
**LDH U/L**	217 (130)	337 (388)	138 (41.3)	172 (112)
**Fibrinogen mg/dL**	434 (80.2)	742 (431)	819 (252)	772 (193)
**PT %**	**89 (11.8)**	**75 (4.36)**	78.7 (12.6)	70.0 (20.3)
**M-EASIX**	3.13 (4.15)	89.4 (142)	259 (174)	680 (658)
**EASIX Score**	**3.64 (3.87)**	**11.5 (0.85)**	14.0 (13.1)	38.6 (35.9)

NETs-IC group, group of patients with a negative NETs induction capacity; NETs+IC group, group of patients with a positive NETs induction capacity Hb, hemoglobin; RDW, red cell distribution width; MPV, mean platelet volume; CRP, C-reactive Protein; Cr, creatinine; PDW, platelet distribution width; NRL, neutrophil-to-lymphocyte ratio; PT, prothrombin time. Data were expressed as mean (± standard deviation). Bold values indicate a statistical significance (p<0.05).

### 3.6 NET induction capacity significantly correlates with the EASIX score and was related to inflammatory markers and non-myeloablative conditioning regimen

In our cohort, NETosis was not associated with sex, age, donor type, stem cell source or HLA identity (*P* > 0.05). We also assessed hematological and serological parameters and correlated them with NETosis. Before conditioning settings (D-1) we found a positive correlation between the EASIX score, a surrogate marker of inflammation and morbimortality, and the NETsIC of allo-HSCT patients samples (p<0.014. r=0.5) (**Figure 4G**). In fact, the EASIX score was significantly higher in patients with a strong NETsIC (p<0.05) (**Table 5**). Moreover, the subgroup of patients with a strong NETsIC exhibited a more compromised hematological profile characterized by significantly diminished hemoglobin levels (p<0.05) and reduced platelet counts (p<0.05) (**Table 5**).

It is well known that conditioning regimens damage host tissues and cause the release of inflammatory mediators. Thus, we also evaluated hematological and serological parameters at D5 after graft infusion. Conversely to D-1, the EASIX score was not significantly correlated with NETsIC. However, the M-EASIX score, which replaces creatinine with CRP, was significantly higher in the NETs+IC group compared to the NETs-IC group (**Table 4**). In addition, the inflammatory markers CRP and IL-6, as we mentioned above, were higher in the NETs+IC group compared to the NETs-IC group, suggesting a pro-inflammatory state in patients with a positive NETsIC (**Tables 3**, **4**). Furthermore, at D10 after transplant, allo-HSCT patients with a strong NETsIC continued having low platelets counts (p<0.05) as well as higher mean platelet volume (p<0.05) and neutrophil-to-lymphocyte ratio (p<0.05) (**Table 5**).

Surprisingly, patients who underwent an RIC regimen presented a higher capacity to induce NETosis than patients on other conditioning regimens. Specifically, 78% of plasma samples from patients who underwent non-myeloablative conditioning presented a positive NETsIC (p<0.05) (**Table 6**).

**Table 6:** Percentage of global GVHD and conditioning treatment type of patients with a negative (NETs-IC) and positive (NETs+IC) capacity to induce NETs formation.

Global GVHD	D-1		Conditioning	D5	
	NETs-IC group	NETs+IC group		NETs-IC group	NETs+IC group
NO	71.4%	33.3%	RIC	20%	78.60%
YES	28.6%	66.7%	MAC	80%	21.40%

NETs-IC group, group of patients with a negative NETs induction capacity; NETs+IC group, group of patients with a positive NETs induction capacity. RIC, reduced intensity conditioning; MAC, Myeloablative conditioning.

### 3.7 NETsIC as bio-markers of clinical severity

A substantial proportion of patients showed severe complications after allo-HSCT (**Table 2**). For that reason, we evaluated hematological, biochemical parameters and NETsIC in patients according to the presence of severe complications (**Table 2**).

**Figure 5:**
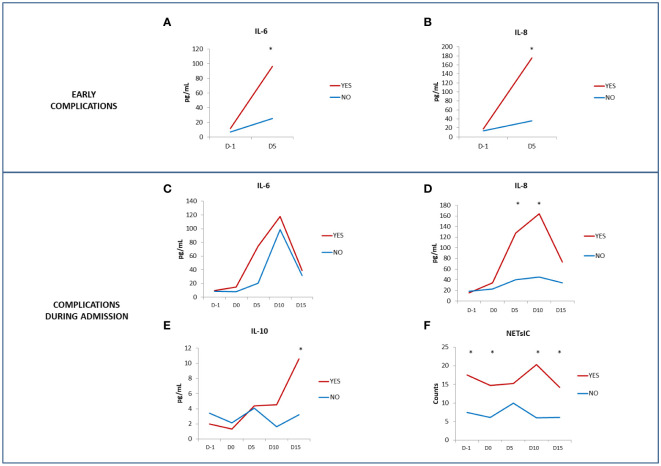
Serum cytokine levels and presence of severe complications. Comparison of IL-6 **(A)** and IL-8 **(B)** levels (pg/ml) between patients who showed early severe complications (red line) and patients who did not (blue line). Comparison of IL-6 **(C)**, IL-8 **(D)** and IL-10 **(E)** levels (pg/ml) and NETsIC **(F)** between patients who presented complications during admission (red line) and patients who did not (blue line). (T-test and ANOVA tests p-values: *p<0.05).

Interestingly, patients who developed early severe complications had, at D5 after transplant, significantly higher IL-6 and IL-8 levels compared to patients without complications in that period (95.91 pg/ml vs 25.02 pg/ml, p<0.01; 205.8 pg/ml vs 36.23 pg/ml; p<0.05; respectively) (**Figures 5A, B**).

Note that, as depicted in **Table 2**, only 16% of patients did not show complications during admission.

Despite the limited sample size, we found before (D-1), during (D0) and after transplant (D10 and D15) a higher NETsIC in patients who presented complications during admission compared to patients without them (**Table 7** and **Figure 5F**). Patients with complications during admission also had higher levels of IL-8 and IL-10 (**Table 7** and **Figures 5D, E**). Moreover, previous to the conditioning regimen (D-1) there is a trend toward a higher percentage of patients who developed GVHD either during or after admission in the NETs+IC group (p=0.1). Specifically, 66.7% of the patients who developed GVHD during or after admission (during follow-up in ambulatory care) had a positive NETsIC (**Table 6**).

**Table 7:** Comparison of total NETs counts, serum cytokine levels and the EASIX and M-EASIX scores between patients who showed complications during admission and patients who did not.

Complications during admission														
	D-1		D0		D5		D10				D15			
	No	Yes	No	Yes	No	Yes	No		Yes		No		Yes	
NETs counts	**7.50 (3.77)**	**17.5 (13.3)**	**6.17 (3.75)**	**14.8 (10.2)**	10.0 (8.66)	15.3 (8.58)	**6.0 0(5.57)**		**20.3 (16.1)**		**6.17 (4.25)**		**14.2 (9.10)**
IL-8 (pg/mL)	18.1 (3.25)	15.6 (8.81)	23.0 (5.94)	34.5 (48.3)	**39.8 (27.8)**	**152 (202)**	**45.5 (32.8)**		**166 (143)**		34.2 (30.0)		73.7 (103)
IL-6 (pg/mL)	8.67 (7.63)	9.70 (9.84)	8.19 (3.65)	14.7 (18.3)	20.6 (6.91)	74.6 (95.6)	98.8 (103)		118 (99.3)		31.8 (42.8)		38.8 (35.4)
IL-10 (pg/mL)	3.42 (3.84)	2.00 (2.07)	2.17 (1.90)	1.34 (0.93)	4.07 (3.55)	4.40 (5.33)	1.62 (1.07)		4.56 (3.51)		3.22 (3.02)		10.6 (28.8)
EASIX Score	**1.60 (1.14)**	**5.50 (4.77)**	**2.60 (1.90)**	**11.0 (11.8)**	18.0 (20.2)	26.2 (41.2)	13.3 (13.6)		25.6 (28.4)		25.7 (34.5)		19.3 (23.4)
M-EASIX Score	2.81 (4.70)	19.4 (62.5)	19.4 (19.9)	37.4 (44.5)	241 (219)	320 (417)	196 (186)		468 (493)		327 (378)		420 (805)

Data were expressed as mean (± standard deviation). Bold values indicate a statistical significance (p<0.05).

Although there were no differences in IL-6 levels (**Figure 5C**), 2 patients who died during the study, after D5 (septic shock and cerebral bleeding), presented increased levels of IL-6 at D5 after transplant thus, in this small cohort, IL-6 levels correlated with overall mortality (p<0.05) (data not shown).

Regarding to EASIX score, previous to the conditioning regimen (D-1) there is a trend toward higher EASIX score in patients who developed GVHD either during or after admission (p=0.095) (data not shown). Furthermore, when we take into account complications during the patient’s whole admission the EASIX score at D-1 and D0 could predict overall clinically significant complications (**Table 7**). Interestingly the M-EASIX score, at D0 was able to predict early complications before day 15 (12.3 vs 50.8; p=0.026) but lacked prediction capability for overall complications during admission in all time points (**Table 7**).

## 4 Discussion

Factors predicting post-allo-HSCT morbidity are not well-known. There is increasing evidence that endothelial dysfunction is involved in many of the life-threatening complications of allo-HSCT (10). Endothelial dysfunction and proinflammatory immune responses could be induced by NETosis (19). The present article delves into the role of NETosis, inflammation and post-transplant complications in allo-HSCT settings. We describe, for the first time to our knowledge, that plasma from patients undergoing allo-HSCT had a higher NETs induction capacity (NETsIC) than plasma from healthy donors and that ability was present throughout the evaluated period (before, during and after transplant). Indeed, NETsIC was related to non-myeloablative conditioning regimen, IL-6, IL-10, CRP, the EASIX score and complications during admission among others.

Prior to allo-HSCT, conditioning therapy is used for disease eradication, the creation of space for engraftment and immunosuppression. Conditioning therapy includes combinations of chemotherapy, radiotherapy and/or immunotherapy; as such, tissue and endothelial injury is a common secondary effect (20). Non-myeloablative conditioning is designed to be less intensive than ablative regimens, thus avoiding the complete destruction of bone marrow. One of its advantages is that there is a lower risk of severe infections during the recovery phase. In our cohort, higher NETsIC was related to non-myeloablative conditioning. The partial preservation of the immune system including a greater number of host immune cells like neutrophils may be related to this finding. In fact, it is also not clear how many patient neutrophils can survive the conditioning regimen, *in vivo*, and remain in bone marrow or other tissues before the donor engraftment since it can vary widely among patients due to the conditioning regimen used or the individual patient response. Neutrophils, actively contribute to host defense by killing pathogens but they may become a significant source of various proinflammatory cytokines, chemokines and growth factors (21). Microbial, viral stimuli and other proinflammatory mediators, such as cytokines, have been described as NETs inducers (22).

Recipients of allo-HSCT are at high risk for contracting infectious diseases. In this context, several studies reported NETosis impairment in the early weeks of engraftment and even, a persisting partial NET impairment for 7 months after allo-HSCT (23, 24). J. Gleen et al. (24) demonstrated a significant decrease in NET formation by neutrophils, isolated both pre- and post-engraftment. These studies are focused on neutrophil function and their capacity to produce NETs while, in the present work, we have evaluated the capacity of patient’s serum to induce NETosis in healthy control neutrophils. Therefore, we assessed different parameters, and our results are not inconsistent with previous data. These data suggest that although the patient´s neutrophils could be impaired to undergo NETosis themselves, the microenvironment in allo-HSCT patients can promote NETosis.

Multiple NETs inducers including cytokines have been described (14, 15). However, few studies have assessed, in allo-HSCT, the specific interactions between cytokines, NETosis and the immune cells involved before and after the conditioning regimen. In our cohort, NETsIC was only related to the anti-inflammatory cytokines IL-10 and IL-6. Although it seems paradoxical at first glance, in our cohort, IL-10 a known anti-inflammatory cytokine (25) was related to increased NETsIC previous to the conditioning regimen. M. Garley et al. (26) reported how IL-10 can induce NETosis after prolonged exposure in cell culture. In our patient’s real-life situation, the higher IL-10 levels found in allo-HSCT patients with a positive NETsIC could be related to the inflammatory microenvironment of the disease or previous treatments (27), where the IL-10 production is trying to dampen inflammation.

IL-8 and IL-6 levels significantly increase after transplant and patients who had a positive NETsIC had higher IL-6 and CRP levels at day 5 after transplant. IL-6 is a multifunctional proinflammatory cytokine often associated with acute phase responses. IL-6 can upregulate the acute phase reactant CRP, therefore CRP partly reflects IL-6 signaling (28). Both parameters could indicate a heightened immune response and inflammation in the group of patients with a positive NETsIC. Similar results were published by Bonneau et al. (29) that reported higher circulating inflammatory cytokines (IL-6 and IL-8) and NETosis biomarkers in the setting of primary graft dysfunction after lung transplantation. In fact, although NETosis and NET generation are important for preventing pathogen invasion, their excessive formation can result in a slew of negative consequences, such as autoimmunity, inflammation and tissue damage (30–32). Moreover, when NETs regulation is affected and NET formation is activated in the circulation, hypercoagulability and thrombosis can be induced (33). Inflammation can trigger the liver production of fibrinogen, which is also an acute phase reactant and contributes to the clotting response. The increased fibrinogen levels allow a higher availability of substrate for thrombin to convert fibrinogen into fibrin, allowing stable blood clot production. In our cohort, fibrinogen could also play a role as an acute phase reactant and, follows the tendency of low PT in the subgroup of patients with a positive NETsIC. Our results could reflect the already known close relationship between the NETs, inflammation and thrombosis described in several disease models.

EASIX score, a surrogate dynamic marker of inflammation, has been reported to predict NRM when assessed before allo-HSCT (34, 35) and as a predictor of intensive care unit admission (36). In our cohort, the EASIX score, previous to the conditioning setting, was significantly correlated with NETsIC. Moreover, the M-EASIX score, which replaces creatinine for CRP, was higher in the NETs+IC group on day 5 after the transplant. These scores seek to determine inflammation and endothelium damage with easy and accessible laboratory tests. In the present study, we demonstrate that they are related to the plasma’s ability to induce NETs formation, *in vitro*.

In our cohort, early complications were related to high levels of IL-6 and IL-8. These results are in agreement with other studies that have shown that serum levels of certain cytokines are elevated after transplant and they correlate with disease severity (37). Although early complications were not related to NETosis by itself, interestingly, IL-6 at day 5 after transplant was related to NETosis and IL-6 levels correlated with overall mortality. In fact, NETosis could predict overall non-specific but clinically significant complications during the full patient admission but lacked a strong predictive capability for early complications before day 15. EASIX score could also predict complications during the full patient admission but only when measured before the conditioning or on day 0 of the study. Similar to that observed for NETosis, in our cohort, the EASIX score lacked the predictive capability for early complications before day 15, probably due to the limited sample size. Interestingly, the ability of plasma to induce NETosis, *in vitro*, seems to correlate better with overall complications during admission, than the EASIX score.

The M-EASIX at day 0, after the conditioning, could predict early complications before day 15, but not overall complications in our cohort. It’s interesting how the change in the biomarker used influences the model, although it makes sense that creatinine, reflecting kidney function, is associated with overall morbidity and mortality. Michel et al. reported that increased creatinine levels were related to mortality in patients undergoing allo-HSCT admitted to the intensive care unit (38). Instead, the CRP focuses on acute inflammatory response and can reach peak levels around 48 hours after stimuli, making it more sensitive to acute complications. Artz et al. have also reported the utility of CRP, measured before the conditioning regimen, to predict complications and mortality in allo-HCST patients (39).

Our study has some limitations: the results for clinical associations were obtained from a small patient cohort since it’s a single-center study. Another limitation is the fact that training is required for the cytology observers, but we think it is possible to improve and automatize the process in the future through machine learning (40) and it has clear potential to be integrated into routine clinical laboratory practice. One of the strong aspects of the present work is that the neutrophil culture assay does not require the isolation of individual patient neutrophils, allowing flexibility of use in clinical practice.

In summary, we found that NET formation could be directly induced by plasma from allo-HSCT patients and was associated with clinically relevant indicators of disease severity, cytokines levels and inflammatory markers. The association with cytokines or other factors in the serum has not been assessed in those settings and could be of interest to understanding the complex mechanism of NETosis during allo-HSCT. The assessment of cytokines (IL-6 and IL-8) and the capacity of plasma samples to induce NETosis, which relates to inflammation, could add value to the EASIX and M-EASIX scores and could also predict patients with a higher risk of non-relapse mortality. In fact, in our cohort, the ability of plasma to induce NETosis, *in vitro*, seems to correlate better with overall complications during admission than EASIX or M-EASIX score and could be of great use.

Further investigations are required to identify the specific factors in the plasma of allo-HSCT patients that may be responsible for inducing NETosis, a better understanding of these factors could help us define the grey line between beneficial and pathological NETosis.

## Data availability statement

The raw data supporting the conclusions of this article will be made available by the authors, without undue reservation.

## Ethics statement

The studies involving humans were approved by Balearic Islands Clinical Research Ethics Committee. The studies were conducted in accordance with the local legislation and institutional requirements. The participants provided their written informed consent to participate in this study.

## Author contributions

BL: Conceptualization, Data curation, Formal analysis, Funding acquisition, Investigation, Methodology, Writing – original draft, Writing – review & editing, Project administration, Resources. VC: Conceptualization, Formal analysis, Investigation, Methodology, Supervision, Writing – original draft, Writing – review & editing, Project administration. VA: Data curation, Investigation, Methodology, Writing – review & editing. LB: Data curation, Writing – review & editing. MS: Data curation, Investigation, Writing – review & editing. AM: Data curation, Investigation, Writing – review & editing. JI: Writing – review & editing. MJ: Formal analysis, Writing – review & editing. MD: Writing – review & editing. MB: Writing – review & editing. JM: Formal analysis, Software, Writing – review & editing. AS: Supervision, Writing – review & editing.

## Supplementary material

Supplementary Table 1 Comparison of total NETs counts and median cytokine levels (pg/mL) between treated and untreated patients previous to the conditioning regimen. Data were expressed as mean (± standard deviation).
