# Supplementary material for: Plasma from patients undergoing allogeneic hematopoietic stem cell transplantation promotes NETOSIS in vitro and correlates with inflammatory parameters and clinical severity
Source: Front Immunol. 2024 Mar 14;15:1353106. doi: 10.3389/fimmu.2024.1353106 (PMC10972998; doi:10.3389/fimmu.2024.1353106)
Supplement: Supplementary Table 1 — Comparison of total NETs counts and median cytokine levels (pg/mL) between treated and untreated patients previous to the conditioning regimen. Data were expressed as mean (± standard deviation). [file Table_1.docx]

**Supplementary table 1.** Comparison of total NETs counts and median cytokine levels (pg/mL) between treated and untreated patients previous to the conditioning regimen.

|  | **D-1** | | **D5** | |
| --- | --- | --- | --- | --- |
|  | **Untreated**  **patients** | **Treated**  **patients** | **Untreated**  **patients** | **Treated**  **patients** |
| **NETs counts** | 11.9 (11.4) | 17.0 (13.3) | 15.2 (6.13) | 14.2 (9.30) |
| **IL-8 (pg/mL)** | 13.7 (8.40) | 16.6 (8.25) | 66.6 (63.3) | 152 (209) |
| **IL-6 (pg/mL)** | 15.2 (13.2) | 8.03 (7.95) | 119 (160) | 52.1 (62.0) |
| **IL-10 (pg/mL)** | 1.26 (1.32) | 2.48 (2.52) | 2.29 (1.86) | 4.89 (5.47) |
| **TNFα (pg/mL)** | 0.34 (0.18) | 0.43 (0.51) | 0.13 (0.13) | 0.25 (0.29) |

Data were expressed as mean (± standard deviation).
